# Single stage reconstruction of a neglected open book pelvic injury with bladder herniation into the upper thigh: a case-report

**DOI:** 10.1007/s00402-020-03555-8

**Published:** 2020-07-29

**Authors:** Michiel Herteleer, Joachim Thüroff, Pol Maria Rommens

**Affiliations:** 1 Zentrum für Orthopädie und Unfallchirurgie, Universitätsmedizin Mainz, Langenbeckstraße 1, 55131 Mainz, Germany; 2 Katholisches Klinikum Mainz, An der Goldgrube 11, 55131 Mainz, Germany

**Keywords:** Pelvic fracture, Open book, Malunion, Reconstructive trauma surgery

## Abstract

When open-book injuries are neglected and result into a pelvic malunion or nonunion, long-term problems, such as chronic pain, gait abnormalities, sitting discomfort, neurological symptoms and urogenital symptoms can occur. In this case report, we describe the repair of a neglected pelvic disruption with the dislocation of the urinary bladder in a one-stage procedure. The clinical image with which the patient presented could be split into unique sub-problems, for which separate solutions needed to be chosen: large symphysis diastasis, instability and pain in both SI joints, malunion of the superior and inferior pubic rami fractures; and urinary bladder herniation into the upper thigh. In a single-stage procedure, the pelvic ring was reconstructed and the bladder reduced. The patient was thereafter continent for urine and could walk independently. A complex clinical problem was divided into its sub-problems, for which specific solutions were found.

## Introduction

Open-book fractures are the result of a high-energy antero-posterior energy transfer on the pelvic ring. In rare cases, a low-energy trauma can also cause an open-book-type injury [1]. When this injury is neglected and results into a pelvic mal- or nonunion, long-term problems, such as chronic pain, gait abnormalities, sitting discomfort, neurological symptoms and urogenital symptoms, can occur [2]. Delayed, reconstructive interventions that aim to restore congruity of the pelvic ring may lead to improved clinical outcomes [2–4]. However, these interventions are challenging as the pelvic mal- or nonunion often has changed the location and function of intrapelvic organs and nearby joints. Consequently, restoration of anatomy and stability may need a multidisciplinary approach and can involve consecutive operative procedures [3, 4].

In this case report, we describe the repair of a neglected pelvic disruption and the dislocated bladder in a one-stage procedure. We especially focus on the preoperative analysis and decision-making of such a complex problem.

## Case presentation

### History, evaluation and preoperative planning

A 65-year-old female patient, from abroad, was referred to our outpatient clinic 5 years after having sustained an accidental split that resulted into an open-book pelvic injury. The injury was primarily treated with an external fixator, which was removed after 6 weeks. During the rehabilitation phase, she suffered a recurrent diastasis of the pubic symphysis with urinary incontinence. Both conditions were further treated conservatively.

**Fig. 1:**
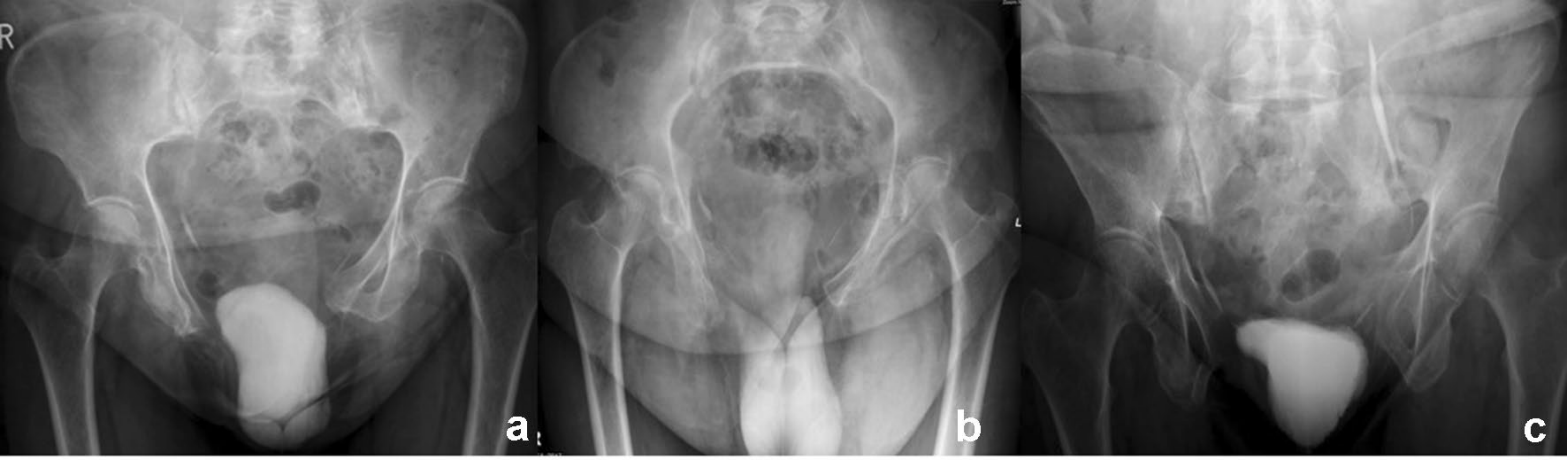
Pre-operative conventional X-rays with simultaneous cystogram. From left to right: AP view (**a**), inlet view (**b**), outlet view (**c**). The malunion of the pubic rami is best seen in the AP view, (**a**) the bladder herniation in the inlet view (**b**)

**Fig. 2:**
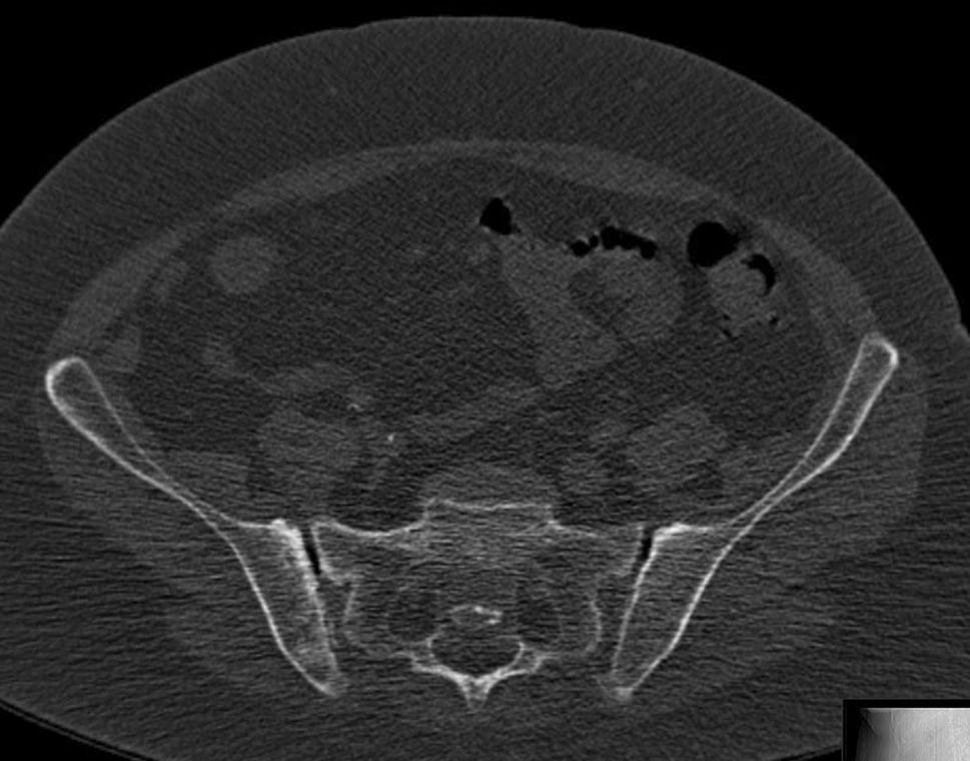
CT-slice through the posterior pelvis. CT evaluation allows for adequate visualization of the SI joint and sacrum. In this image widening of both SI joints can be seen with bilateral signs of degenerative SI joint arthrosis. The typical cortical densening can be best seen on the iliac side of the SI joint

Her walking distance was very limited, her walking pattern was uncertain, painful at the lower back and with exorotated lower extremities. There was a large soft-tissue hernia at the place of the pubic diastasis. The conventional X-rays (a.-p. pelvic, inlet and outlet views) with cystography showed a severe pubic diastasis without a vertical displacement of one of the hemipelves. There was a malunion of the right superior and inferior pubis ramus in exorotation. The cystogram showed a dislocated bladder descending through the pubic diastasis into the left medial upper thigh (Fig. 1a–c). The preoperative pelvic CT with cystogram confirmed the herniated bladder and severe pubic diastasis. Both iliosacral joints showed moderate signs of arthrotic degeneration (Fig. 2). The medical history of the patient included hypertension, COPD and Addison’s syndrome. She was classified as an ASA III–IV patient by our anesthesiologists.

### Preoperative analysis and decision-making

The plurifactorial problem of the patient was divided into several sub-problems, for which a separate solution had to be found: (1) severe pubic diastasis (2) in exorotation malunited right superior and inferior pubic ramus (3) arthrotic degeneration of both iliosacral joints (4) urinary bladder herniation into the left upper thigh. The following procedures were chosen for the solution of each specific problem: (1) the pubic diastasis needed an open reduction and internal fixation, (2) the malunited pubic rami fractures needed a correction through osteotomy, (3) the arthrotic iliosacral joints needed debridement and arthrodesis; (4) and the dislocated bladder needed mobilization, reduction and stable fixation in its anatomical position. As the solutions of these problems could not be carried out in separate operative procedures, the decision for operative repair in a one-stage procedure was made.

### Surgical management

A stepwise approach within one operative procedure was performed to consecutively and adequately treat the abovementioned problems.

The operation started with exposure of the iliosacral joints through the lateral window of the ilioinguinal approach on both sides The SI joints were debrided and the joint spaces filled with cancellous bone grafts from the iliac crests. Two 3-hole bended stainless steel plates were used on each side to stabilize the SI Joints. On the sacral side of each plate, one long cancellous screw was used, on the iliac side two shorter cortical screws.

The consecutive part of the procedure was performed through a Pfannenstiel incision of about 20 cm of length. The urinary bladder was exposed carefully and reduced into the small pelvis. Through cystoscopy, a double J catheter was placed in each ureter for better identification and prevention of surgical damage. A hysterectomy was performed, which enabled attaching the roof of the bladder directly to the promontorium with three non-resorbable single stitches (Ethibond-0, 5/8 needle), which were placed with 2 cm distance from each other. With a polypropylene mesh, the vaginal stump was attached to the promontorium as well.

**Fig. 3:**
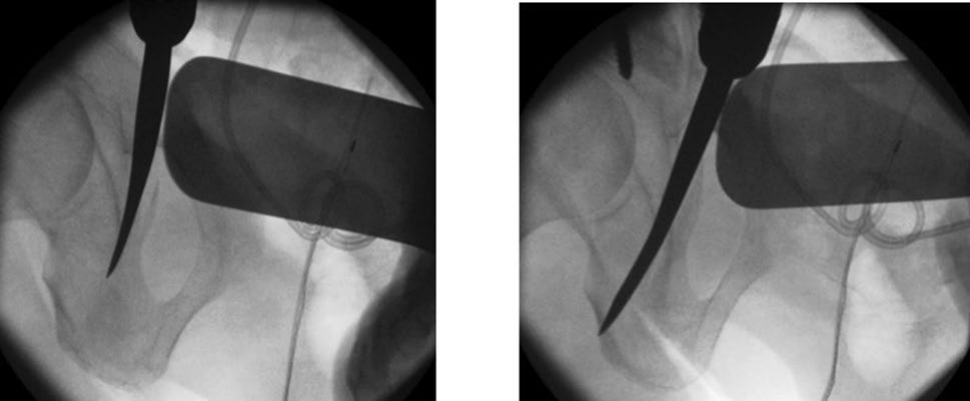
Osteotomy of the superior and inferior pubic rami with a chisel. It is recommended to perform the osteotomy under fluoroscopic guidance as care must be taken not to perforate the hip joint or injure the obturator nerve

**Fig. 4:**
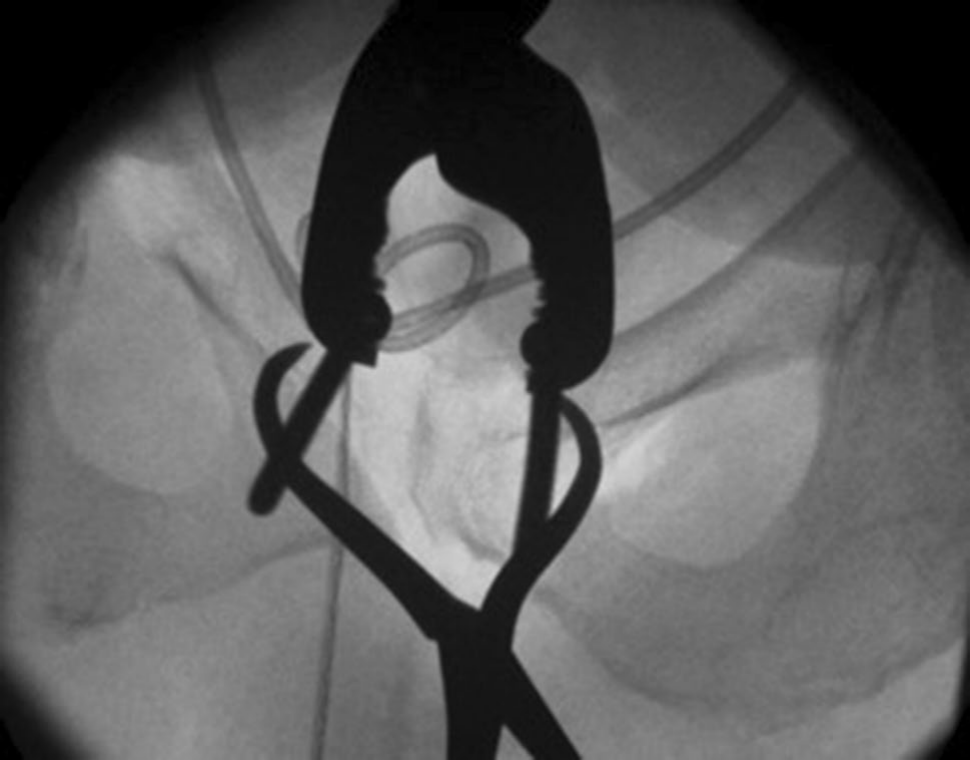
Closure and double plating of the symphysis after reduction with Farabeuf clamp and reduction forceps. With the Farabeuf clamp a large amount of force can be used to reduce the pelvis. Once it is reduced the reduction forceps holds the reduction in place so that the Farabeuf clamp and screws can be removed and the superior plate can be placed and fixated over the symphysis pubis

**Fig. 5:**
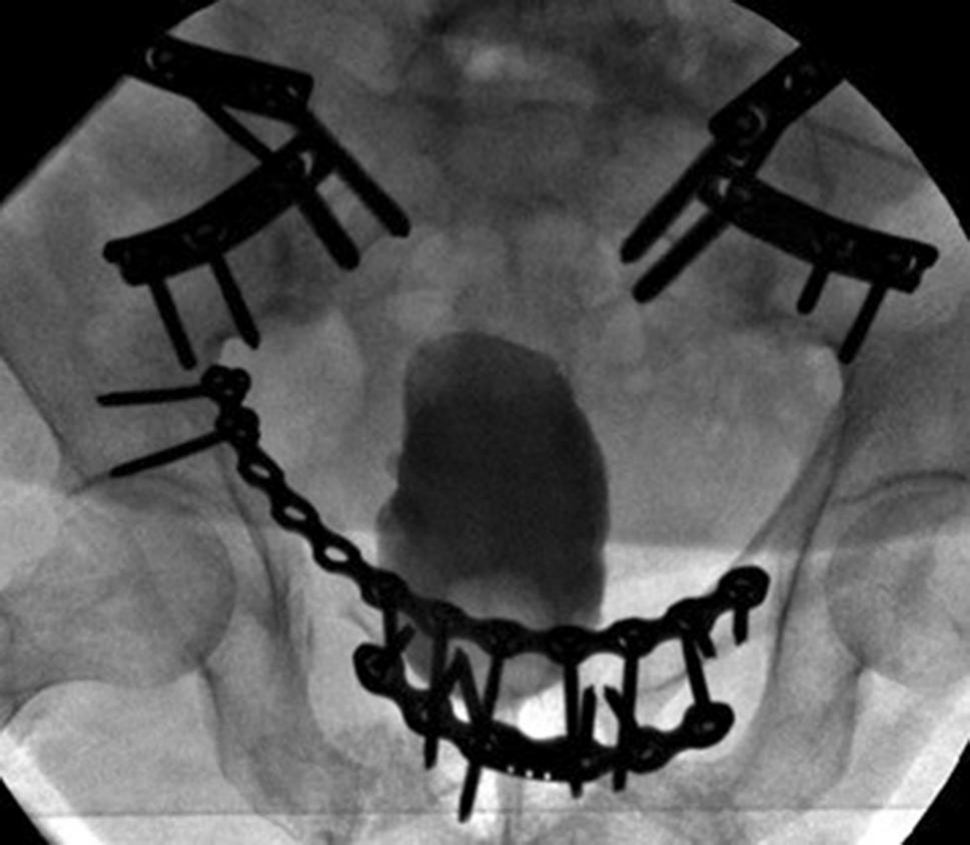
Intraoperative AP X-ray of the pelvic ring with cystogram. The bladder is back into its anatomical position

The third part of the intervention consisted of an osteotomy of the exorotated right superior und inferior pubic ramus (Fig. 3). Due to the malunion, the symphysis could not be closed adequately without osteotomy. After the osteotomy, the pubic symphysis could be closed with the help of a Farabeuf clamp (Fig. 4). The reduced symphysis was finally stabilized with a double-plate osteosynthesis. A long 12-hole reconstruction plate was placed over the symphysis and right superior pubic ramus, extended over the performed osteotomy until it reached the supraacetabular area. A second, shorter angular stable six-hole plate was placed anteriorly. An intra-operative retrograde cystography was carried out and showed an adequate position of the bladder in combination with a complete restoration of the pelvic ring (Fig. 5).

### Postoperative care

**Fig. 6:**
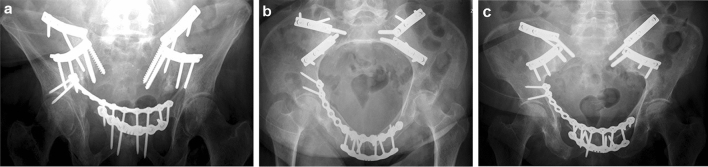
Post-operative conventional X-rays. From left to right: AP view (**a**), inlet view (**b**), outlet view (**c**). End result of the intervention. The SI joints posteriorly are debrided and fixed. The symphysis is double plated anteriorly, the superior plate extends over the osteotomies of the right ischio- and ilio-pubic rami and the pelvic ring is closed

Both double J ureteral stents and the bladder catheter were removed after 18 days. After removal, the patient had a normal urinary function without incontinence. During the in-patient rehabilitation, a seroma in the old hernial sac was noticed. A surgical evacuation was performed after 31 days. Fluid cultures were positive and a re-intervention with application of a vacuum dressing was performed at day 41 and intravenous antibiotics were administered. The vacuum dressing was removed at day 51 and the wound closed secondarily. Postoperative course was further uneventful. At the day of discharge, the patient could walk pain-free with aids. After discharge, the patient returned to her country of residency where she initially stayed at the referring University Hospital and then in a rehabilitation clinic. She returned home 3 months after leaving our hospital. She never came back to our center for clinical follow-up, but we had contact with the referring colleague until 5 years after the procedure. Two years after the intervention, a CT scan was performed which showed fusion of the SI Joints and a completely healed pelvic ring. No signs of implant loosening were seen. The patient had no pain in the SI regions and she could walk undisturbed (Fig. 6a–c). She did not wish to have the implants removed. The patient followed a training program for better control of urinary continence. Although there was still some incontinence, there has been a significant amelioration in comparison to the time before pelvic surgery. The patient has no sexual life anymore.

Due to her previous illnesses and her long-term restricted mobility before the pelvic correction, the patient has low functional demands. She is able to perform activities of daily living (washing herself, dressing and undressing), walking and shopping in the city, etc. She is a housewife and has no sports activities. At home, she has continuous assistance for her household. Since her discharge from our hospital, the patient was hospitalized four times at hospitals in her country of residence: two times for meningitis, for which she had craniotomy once, one time for spinal stenosis and one time for pneumonia.

## Discussion

Open-book injuries can occur due to two different mechanisms. The most common mechanism is an antero-posterior compression force which disrupts the pelvic ring anteriorly. A less common mechanism is a forced abduction and exorotation of one leg [1, 5]. An open-book lesion is characterized by a rupture of the symphysis with a uni- or bilateral opening of the SI joints. The strong posterior SI ligaments remain intact resulting in a rotational but not a vertical instability. In our patient, an accidental split caused the injury [1, 5]. The initial treatment was insufficient; the obtained stability was low and the fixation period was short. Consequently, recurrent diastasis occurred after fixator removal.

Due to continuing walking, the iliosacral joints were overstressed and degenerative arthrosis developed over time (Fig. 2). Arthrodesis of the sacroiliac joints helped to restore stability of the posterior pelvic ring and significantly diminished low back pain [6]. Anterior approaches to the SI joints were chosen. These approaches were possible with the patient in the supine position and enabled a debridement of the joints and direct arthrodesis with intra-articular bone grafts and double bridging plate osteosynthesis. For the consecutive phases of the operation, the patient could remain in the supine position.

Closure of the anterior pelvic ring would not have been possible without osteotomy of the superior and inferior pubic rami. It is recommended to perform the osteotomies at the site of the malunion as this normally makes it easier to restore the anatomy of the pelvic ring [2]. In this case, the surgeon needs to be well aware of the proximity of the hip joint and the obturator nerve and take care not to damage these structures. (Fig. 3). For maximal stability during the healing period and prevent failure, a double-plate osteosynthesis of the anterior pelvic ring was chosen. [7–9]. Closure of the pubic symphysis was not possible without reduction of the dislocated bladder. To prevent recurrence of bladder herniation and urine incontinence, a direct attachment to the promontorium with several stitches and polypropylene mesh implantation was performed. This procedure is recommended in severe cases of stress urinary incontinence with organ prolapse [10]. As all parts of the operation could not be separated from each other, the procedure was done in one narcosis.

The double-plate osteosynthesis was meant for accurate reduction and stable fixation of the joint. In this way, a durable scar tissue formation can take place. By leaving the implants in place, a recurrent opening of the symphysis is avoided, but a limited (physiologic) movement of the joint was allowed. Fusion of the joint would have little chance of healing due to two reasons: (1) Lack of enough bone grafts of good quality (2) The fusion of both SI joints would have put additional shear stresses on the symphysis while walking, preventing bony healing of the fusion.

A neglected open-book injury in combination with a herniation of the bladder in the medial upper thigh is an extremely rare condition. The solution of this complex problem exists in its division into several sub-problems, for each of which a specific solution is chosen. In this case, all problems had to be solved in one operative procedure, because the sub-problems were interconnected and could not be solved separately.

Post-traumatic pelvic deformities are usually treated in multiple-stage procedures [3, 4, 11]. This case shows that it is possible to treat these deformities in a single-stage procedure if the patients’ physiological status allows it. Due to the long operative procedure, there is a higher risk of infection of the surgical site. Fortunately, the postoperative infection in this patient was restricted to the herniation sac in the upper thigh. Debridement and vacuum therapy were sufficient to control the infection. Debridement of the small pelvis and removal of implants were not necessary.

## Conclusion

Malunions of pelvic ring injuries are rare. To restore the malunion, several operative procedures are necessary. This case shows that it is possible to treat a severe malunion with concomitant herniation of the urinary bladder in one operation. The key to success is to separate a complex clinical problem into separate problem entities. For each single problem, a solution is chosen and consecutively performed.
