# Correction to: Single stage reconstruction of a neglected open book pelvic injury with bladder herniation into the upper thigh: a case-report

**DOI:** 10.1007/s00402-021-04013-9

**Published:** 2021-07-13

**Authors:** Michiel Herteleer, Joachim Thüroff, Pol Maria Rommens

**Affiliations:** 1 Zentrum für Orthopädie und Unfallchirurgie, Universitätsmedizin Mainz, Langenbeckstraße 1, 55131 Mainz, Germany; 2 Katholisches Klinikum Mainz, An der Goldgrube 11, 55131 Mainz, Germany

## Correction to: Archives of Orthopaedic and Trauma Surgery (2021) 141:855–859 10.1007/s00402-020-03555-8

The article Single stage reconstruction of a neglected open book pelvic injury with bladder herniation into the upper thigh: a case-report, written by Michiel Herteleer, Joachim Thürof and Pol Maria Rommens was originally published Online First without Open Access. After publication in volume 141, issue 5, page 855–859 the author decided to opt for Open Choice and to make the article an Open Access publication. Therefore, the copyright of the article has been changed to © The Author(s) 2021 and This article is licensed under a Creative Commons Attribution 4.0 International License, which permits use, sharing, adaptation, distribution and reproduction in any medium or format, as long as you give appropriate credit to the original author(s) and the source, provide a link to the Creative Commons licence, and indicate if changes were made. The images or other third party material in this article are included in the article’s Creative Commons licence, unless indicated otherwise in a credit line to the material. If material is not included in the article’s Creative Commons licence and your intended use is not permitted by statutory regulation or exceeds the permitted use, you will need to obtain permission directly from the copyright holder. To view a copy of this licence, visit http://creativecommons.org/licenses/by/4.0/.

The original article has been corrected.

Open Access funding enabled and organized by Projekt DEAL.

